# Healthcare Workers From Diverse Ethnicities and Their Perceptions of Risk and Experiences of Risk Management During the COVID-19 Pandemic: Qualitative Insights From the United Kingdom-REACH Study

**DOI:** 10.3389/fmed.2022.930904

**Published:** 2022-07-01

**Authors:** Irtiza Qureshi, Mayuri Gogoi, Fatimah Wobi, Jonathan Chaloner, Amani Al-Oraibi, Osama Hassan, Daniel Pan, Laura B. Nellums, Manish Pareek

**Affiliations:** ^1^Lifespan and Population Sciences, School of Medicine, University of Nottingham, Nottingham, United Kingdom; ^2^Department of Respiratory Sciences, University of Leicester, Leicester, United Kingdom; ^3^Department of Infection and HIV Medicine, University Hospitals of Leicester NHS Trust, Leicester, United Kingdom

**Keywords:** risk, ethnicity, healthcare workers, safety, COVID-19, risk assessment, PPE

## Abstract

**Introduction:**

Healthcare workers (HCWs) are at higher risk of being infected with severe acute respiratory syndrome coronavirus 2 (SARS-CoV-2). Previous studies have examined factors relating to infection amongst HCWs, including those from ethnic minority groups, but there is limited data regarding the lived experiences of HCWs in relation to self-protection and how they deal with SARS-CoV-2 infection prevention. In this study, we presented data from an ethnically diverse sample of HCWs in the United Kingdom (UK) to understand their perceptions of risks and experiences with risk management whilst working throughout the COVID-19 pandemic.

**Methods:**

We undertook a qualitative study as part of the United Kingdom Research study into Ethnicity and COVID-19 outcomes among Healthcare workers (United Kingdom-REACH) conducting semi-structured interviews and focus groups which were recorded with participants’ permission. Recordings were transcribed and thematically analyzed.

**Findings:**

A total of 84 participants were included in the analysis. Five broad themes emerged. First, ethnic minority HCWs spoke about specific risks and vulnerabilities they faced in relation to their ethnicity. Second, participants’ experience of risk assessments at work varied; some expressed satisfaction while many critiqued it as a “tick-box” exercise. Third, most participants shared about risks related to shortages, ambiguity in guidance, and inequitable distribution of Personal Protective Equipment (PPE), particularly during the start of the pandemic. Fourth, participants reported risks resulting from understaffing and inappropriate redeployment. Finally, HCWs shared the risk mitigation strategies which they had personally employed to protect themselves, their families, and the public.

**Conclusion:**

Healthcare workers identified several areas where they felt at risk and/or had negative experiences of risk management during the pandemic. Our findings indicate that organizational shortcomings may have exposed some HCWs to greater risks of infection compared with others, thereby increasing their emotional and mental burden. Ethnic minority HCWs in particular experienced risks stemming from what they perceived to be institutional and structural racism, thus leading to a loss of trust in employers. These findings have significance in understanding staff safety, wellbeing, and workforce retention in multiethnic staff groups and also highlight the need for more robust, inclusive, and equitable approaches to protect HCWs going forward.

## Introduction

In early 2020, the coronavirus disease 2019 (COVID-19) pandemic had spread rapidly in the United Kingdom (UK). Healthcare workers (HCWs) are known to be at higher risk of infection and worse clinical outcomes such as intensive care admission and death (1). For example, in its earliest phases, a third of all COVID-19 cases in China were in HCWs (2). In June 2020, nearly 20% of Spain’s health workforce had been infected with COVID-19, and 70 HCWs had died (3). Research conducted in the United Kingdom also found that HCWs were 7 times more likely to have severe COVID-19 as compared with those working in “non-essential” jobs (4). Furthermore, ethnic minority HCWs in the United Kingdom were disproportionately affected by the pandemic. Previous research showed that HCWs of Black ethnicity in one large National Health Service (NHS) hospital trust were over 2 times more likely to be infected than non-Black HCWs during the first wave (5). Nationally, ethnic minority HCWs were also reported to account for 63% of overall deaths of HCWs, 64% of nursing and support staff, and 95% of medical staff deaths during that period (6).

The high risks experienced by HCWs triggered a need for staff protection at workplaces, and protection measures such as risk assessments were initiated in the United Kingdom. In April 2020, the NHS advised all its employers and leaders to conduct risk assessments for staff at potentially greater risk (including ethnic minority staff) and make appropriate arrangements to mitigate their increased risk (7). However, a survey conducted by the British Medical Association (BMA), among its members in December 2020, showed that only 46% of its ethnic minority members said that they have risk assessed and felt confident that appropriate adjustments had been made based on the outcomes (8). Existing templates for conducting risk assessments for HCWs have also been criticized as inadequate and failing to take necessary risk factors into consideration while calculating risk scores (9).

The use of Personal Protective Equipment (PPE) as a measure of protecting HCWs was also initiated. However, the availability of PPE became a major concern in the early days of the pandemic, and reports of hoarding, misuse, over-pricing, and delay were rife, thereby putting HCWs at risk (10). In the United Kingdom, PPE shortage was embroiled in a political controversy with the government denying shortage at any time, while ground reports suggested otherwise (11). Apart from the supply, guidelines on PPE usage also varied considerably in the United Kingdom health settings and were at times even incongruent with international guidelines, creating confusion and anxiety among HCWs (12, 13).

Despite the acknowledgment that HCWs are at higher risk, empirical evidence showing how HCWs perceived and experienced these risks are limited. More information is needed on this subject as HCWs should be appropriately supported and protected from mental harm during the pandemic. Previous studies have highlighted how the health workforce emerges as a fundamental part of how health systems have responded to the cumulative challenges posed by the pandemic, concluding that the workforce irrefutably contributes to overall healthcare system resilience (14). The World Health Organization (WHO) has named HCWs as “our most valuable resource for health,” and as they help protect the public, they should also be protected from harm (15). Observational studies have enumerated several factors such as gender, role in patient care, and availability of PPE that put HCWs at risk (16). However, studies exploring lived experiences of HCWs regarding exposure to risk and also the management of risks are scant. Such studies are important to understand the differential risks that different groups of HCWs, particularly those from ethnic minority groups, may have experienced risk and also to know how health system factors influence risk exposure and risk mitigation. In this study, we presented qualitative data collected from ethnically diverse HCWs in the United Kingdom to understand their perception of risks and experiences with risk management, while working during the pandemic. The findings presented in this study will aid the assessment and mitigation of risks faced by HCWs during health emergencies.

## Materials and Methods

### Setting and Recruitment

This study is the qualitative component of the United Kingdom Research study into Ethnicity and COVID-19 outcomes among healthcare workers (United Kingdom-REACH) project, a mixed-method nationwide study initiated to provide rapid evidence on COVID-19 outcomes among HCWs and inform national policy. We aimed to explore the experiences of HCWs during the pandemic. HCWs were defined as clinical and non-clinical staff who aged 16 years and above and working in a healthcare setting (17). HCWs from across the four devolved United Kingdom nations were invited to take part and purposively sampled to include workers from various staff grades, job roles, age, sex, ethnicity, migration status, and United Kingdom nation. Recruitment was through invitation emails sent out *via* NHS trusts, private health contractors, professional bodies, partner organizations, Twitter advertisement, and through the Professional Expert Panel (PEP) and United Kingdom-REACH stakeholder group (STAG). Participant information sheets containing study details were shared with prospective participants before the start of study procedures.

### Data Collection and Analysis

Data collection took place between December 2020 and July 2021. Participants underwent an online consent procedure and supplied demographic data through the same platform. Recruitment was then guided using purposive sampling. Potential participants had the choice to engage in either an interview or a focus group, depending on their choice. Our decision to use both focus groups and individual interviews for data collection was based on the understanding that sharing sensitive information such as experiences of discrimination may be easier for participants in an interview setting. Alternately, we chose the focus group to explore HCW experiences and opinions in a shared environment which would bring out the similarities and differences in experiences and provide greater breadth to the data. Constraints to face-to-face communication led to all interviews and focus groups being conducted remotely *via* Microsoft Teams or telephone (only for interviews). A topic guide was developed in consultation with the PEP and STAG, the public engagement and stakeholder engagement groups, respectively. The topic guide was then piloted with the first eight participants and refined iteratively during data collection where new key issues emerged to ensure it was relevant and current. The final topic guide areas are attached as Supplementary Material. Interviews lasted for 45–60 min and focus groups took approximately 1.5 h, with group sizes varying between 2 and 7 members. Following their participation, a gift voucher was given to HCWs in recognition of their contribution to the research interviews, and focus groups were conducted by FW, MG, AAO, OH, IQ, and LBN, who represent a range of ethnicities, both men and women and are all trained qualitative researchers and culturally competent in working with diverse ethnic groups. Discussions were recorded with permission, transcribed, and anonymized prior to analysis. Further details of methods can be found in our previous publication (17).

As an Urgent Public Health (UPH) study, the study team focused on the rapid generation of evidence, which can inform policy. With this need for expedience in mind, we presented the analysis from 84 participants who took part in 46 interviews and nine focus groups. These transcripts were taken from a larger pool of interviews and focus groups once a data-driven inductive coding approach was adopted to analyze the transcripts (18). The research team (FW, MG, AAO, IQ, JC, and LBN) began with each member reading a certain set of transcripts to build familiarity. Thereafter, FW undertook open coding of the first set of transcripts and identified the preliminary set of codes which was shared with the team. Other members of the team then used the coding framework for coding additional transcripts and regularly updated it in tandem with newly emerging codes. The team developed the final set of themes, once data saturation had been agreed, and through regular discussions and iterations (including checking back themes with participant stakeholder groups) and these are reported as per consolidated criteria for reporting qualitative research (COREQ) guidelines (19).

### Ethics

This study received ethical approval from the London-Brighton and Sussex Research Ethics Committee of the Health Research Authority (Ref No. 20/HRA/4718).

## Results

### Participant Data

**FIGURE 1:**
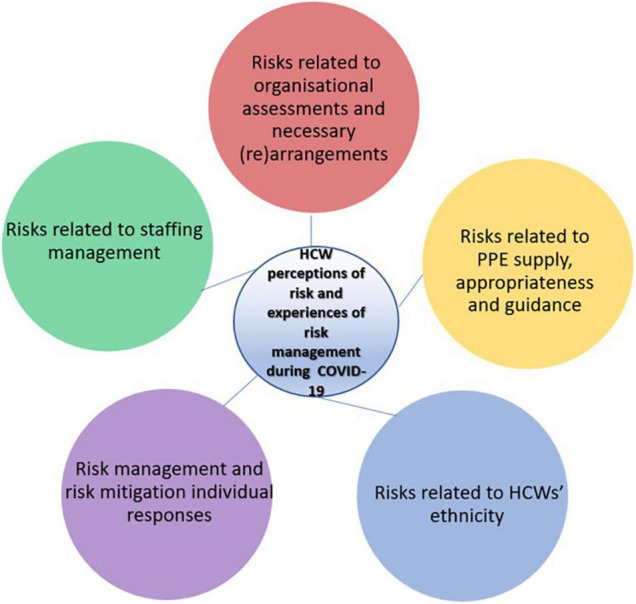
Summary of findings diagram.

This study recruited 84 participants and conducted 46 interviews and nine focus groups with them (refer to Table 1 for participant demographic data). This study includes a detailed analysis of the discussions below (refer to Figure 1 for the summary of findings).

**TABLE 1:** Participants’ demographic data.

Variable	Sample (n = 84)
**Sex**	
Male	28
Female	56
Age (range)	25–53
**Ethnicity**	
Asian∼	33
Black	17
Mixed	08
White	23
Other	03
**Job Role**	
Doctors	18
Nurses and Midwives	18
Allied Health Professionals	32
Administrative and other non-clinical	16
***United Kingdom Region***	
England	72
Scotland	03
Wales	-
Northern Ireland	05
Unknown	04

*∼Asian category includes all those under Asian/Asian British United Kingdom Census categories (Indian/Pakistani/Bangladeshi/Chinese/Other Asian).*

### Risks Related to Healthcare Workers Ethnicity

Some participants commented on the dynamic between their ethnicity and how they experienced risk. This dynamic emerged in narratives across the four other themes identified. However, specific examples included a heightened awareness that people from ethnic minority communities were at greater risk of illness or death:

*I was actually quite worried about getting it because very early on there were signs that people from my sort of community, people of color, were being affected quite a bit and you know, you started seeing these images coming up and they were mainly people that were from an ethnic minority and it did worry me* [P22, Allied Health Professional].

This heightened risk was felt not only by the participants themselves but also by their friends and family:

*So I think in the early stages of the pandemic there was that uncertainty about the difference in risk with your ethnicity and whether people in minority ethnic groups were at higher risk. So I think that did play on my mind a little.*…. *it then sort of plays on your mind a bit more about your family members as well who are also of the same ethnicity and that same higher risk group* [P8, Doctor].

Some participants reported the pervasive impact that their minority ethnic status had on their whole experience of working during the pandemic:

*It’s actually historical that because you’ve got your White privilege, you don’t understand that Black people or people from an ethnic minority are not treated with the same equity, same fairness as you would experience* [P12, Ancillary Health Worker].

This experience is tied into perceptions of discrimination, specifically related to risk responses such as risk assessments:

*You know those attitudes. No-one actually directly said that but you could feel that, you know, you could feel it. As somebody from a BAME* [Black, Asian and minority ethnic]*—you have that sixth sense that people are sort of saying that but maybe not in front of you. And it did—and again the risk assessments (were) slow to come forward* [P12, Ancillary Health Worker].

### Risks Related to Organizational Assessments and Necessary (Re)arrangements

Experiences of COVID-19 risk assessments varied greatly among participants. Not all participants had a risk assessment at work, either because they were not offered or they declined as they perceived themselves to be at low risk. For those who have had a risk assessment, these ranged from informal communications with line managers to meetings with formal documentation. Experience with post-assessment (re)arrangements also varied with most participants saying that nothing changed after their assessments and it was business-as-usual, while a few others said that recommended adjustments were made, for some immediately and for others after several weeks. The differences in risk assessment and responses to staff safety across different teams and workplaces meant that while some participants felt satisfied with these measures, other HCWs were not entitled to the same wellbeing opportunities. As one participant shared:

*I had to email our Trust to say is there going to be a risk assessment? And then when one came out, you know, the initial risk assessment that came out wasn’t very good, it was quite wishy-washy and it didn’t really “yeah, you’re at risk,” then what? It didn’t really say anything else* [P7, Doctor].

Some participants also rued the fact that the risk assessments did not take into consideration their family or living conditions, which, therefore, did not reflect the true nature of risks that they or their families faced. Speaking about it one participant said:

*I was told that your risk assessment is very low, even though your husband is shielding, you’re not the person who’s got the chronic ill health, so you should be absolutely fine*…*my risk was low, because I don’t work on a ward, I’m not very much clinic-based*…*there was no further elaboration on actually living with a family who should be shielding* [P17, Nurse/Midwife].

Furthermore, some participants felt that the consideration of ethnicity was a blanket criterion for risk assessment, which created situations where ethnic minority staff felt they were being judged by their colleagues:

*So then they identified the BAME, that they’re at higher risk therefore we need to do risk assessments—again that made me self-conscious and I felt very uncomfortable with it, because now it’s like “oh right because you’re Asian you’re at higher risk,” “well what about me, I’m White, I’m at risk as well*” [P21, Ancillary Health Worker].

### Risks Related to Personal Protective Equipment Supply, Appropriateness, and Guidance

Having appropriate PPE at work played a major role in participants’ perception of risk. Nearly two-thirds of our participants said that they faced a shortage of PPE in the first wave of the pandemic, and several among them felt that not having enough or appropriate PPE put them at risk. As one participant described the situation with PPE when COVID-19 struck:

*The lack of PPE obviously was a main one as far as risk factors, how much risk were we putting ourselves in, what risks were we bringing back home to our family, what risk were we spreading to our colleagues, you just didn’t know what we were dealing with* [P22, Allied Health Professional].

Even the limited PPE that was supplied during the first wave was not always deemed to be appropriate for their working environments by many participants. They described the porosity of items, the lack of high-grade masks, and policies that allowed the reusing of PPE and usage of expired PPE.

*We are using, or being made to use, expired face masks*…*this happened during the first wave. I noticed that some of the face masks that we were using were a year ago expired. I did let it go that time because during the first wave there’s been a lot of time that we’re running out of supplies. However, this third wave we are still experiencing it and what they’re doing is, on the box they’re covering the expiry date to a new expiration date* [P2, Nurse/Midwife].

The use of PPE prioritization and rationing was also experienced by several participants, and they shared how supply was not equitable across different departments or wards and even staff groups in hospitals. Similarly, compared with hospital-based HCWs, many HCWs working in community settings (e.g., dental surgeries and community pharmacies) reported experiencing PPE shortage, particularly in the beginning, and felt that there was a significant delay in addressing these shortages by the NHS.

*Well the PPE issue, I had problems with some of the nurses in ICU* [Intensive Care Unit] *during the first pandemic. When we domestic go in there for the scrub, they will tell us “no, we’re reserving the ones that we’ve got for our nurses” and I said “but we too are here to work.” I can’t go in ICU with my own uniform. There was a shortage of PPE at that time, they were trying to tell us to go in with the plastic apron* [P21, Ancillary Health Worker].

Participants also reflected on how PPE guidance in relation to face masks and face mask fitting were deprioritized over clinical attention required by patients in the busy working environment. They shared how they felt conflicted to follow instructions, despite feeling they were contributing to unsafe practices and putting themselves at risk:

*I remember there was one time I was on-call and I think I hadn’t been mask fitted yet*… *but I remember it being such a busy shift that my senior was like “it doesn’t matter, can you just go and see the patient,” basically, so it was a high risk patient in resuscitation in A*&*E* [Accident and Emergency]. *I think there was lots of cutting of corners basically, but I remember thinking this is really unsafe, I didn’t feel like I could speak up because I was very junior and I sometimes wish I did but, yeah, I didn’t* [P9, Doctor].

At times, HCWs questioned the motivations behind policy changes and, in turn, changes to local PPE guidelines. Many wondered whether they were in response to the emerging scientific evidence or managerial responses to PPE supply. Participants felt decisions around PPE were ambiguous and changed frequently leaving them vulnerable, unsafe, and pressured to continue service despite feeling inadequately protected:

*It’s the confusion of information coming from the guidelines really that management is giving us. It feels like it’s changing every 5 min during April and—March actually of last year* [2020]. *So we were very confused. Understandably we know it’s a new virus so the guidelines haven’t been set yet so it’s being written but yes, it led to probably a lot of PPE wasted because we were very scared* [P3, Nurse/Midwife].

While PPE shortage and quality were national issues affecting HCWs from all ethnicities, but compared with White British participants (33%), nearly 68% of our ethnic minority participants said that they had faced PPE shortages or discrepancies in the distribution or were using sub-standard PPE. Many of these participants said that the lack of appropriate PPE at work made them feel “*undervalued*” (P24, Doctor), like “*sacrificial lambs*” (P25, Allied Health Professional), and believed they were “*put in harm’s way*” (P5, Nurse/Midwife).

*So it was not surprising that many of the Black, Asian, minority ethnic groups, they were not as assertive as their equivalents because they are the ones that work behind the scenes, they don’t complain, they just do the job, they want the job done. And it is that lack of assertion and inability to ask difficult questions that has allowed many of our members to be exposed to this condition without PPE* (P23, Doctor).

### Risks Related to Staffing Management

Redeployment and staff shielding led to a decline in workforce capacity within both clinical and non-clinical settings, and some HCWs having fewer risk factors felt that they were compelled to take on additional shifts, thereby increasing their chances of exposure and infection. As one participant said:

*Then, with staff going off sick with stress and various things, we had a lot of shortfalls on the ward. So, you were being asked to cover shifts, swap your shifts around, maybe split your shift in half, so you’d cover 2 days, whether you were really needed, rather than working the 1 day and getting out, and so it just felt like you were constantly at work and constantly being asked to work overtime without any break or let up. So, you were just putting yourself more at risk* [P5, Nurse/Midwife].

Some staff also reported that certain people (including ethnic minorities) were more likely to face higher risks such as longer shifts:

*They’re sometimes bank workers, they’re locum [agency] workers, they’re people that work a lot of out of hours shifts, so they work perhaps nights all the time. They’re almost always from ethnic minorities* [P4, Doctor].

Many HCWs also found redeployment challenging. Most who were redeployed had not been working in those roles for some time and felt anxious and worried about their ability to perform efficiently. One redeployed nurse participant shared:

*I will say the second wave was worse than the first one, it was very intense. On the first wave, although I was in intensive care I didn’t actually look after COVID patients*… *on the second time I had to go to* [Place A], *which is a bigger hospital, a bigger unit, I’d never worked there before, I didn’t know anyone, so it was definitely more challenging, much more, it was overwhelming actually. I’ve been a nurse over 20 years and on the first day when I had to wear all the PPE, the hood and I walked into this area, you know, and I saw, it was dark and it was full of COVID patients. And actually I thought I was going to faint because I was so overwhelmed and I’d never felt like that before* [P18, Nurse/Midwife].

Healthcare workers in understaffed teams felt stressed and anxious as they faced critically ill patients with a high risk of greater morbidity and even mortality under their care. Furthermore, high staff absences in specialist areas also meant that specialist clinical activities were often carried out by untrained healthcare colleagues who were the only support available:

*I was asking our managers, our CNSs* [Clinical Nurse Specialists], *to come out of their office because I’m struggling*…*so my colleagues*…*were shielding and I happened to be just running the morning shifts, the 12 h shifts in the mornings. There’s four or five Band 6s* [pay grades indicating relatively experienced nurses] *at night, that’s always been the pattern, and I was overwhelmed and people were dying on our ward and I can see my* [redeployed] *colleagues don’t know what to do and it’s like I want to change my name!* [P3, Nurse/Midwife].

Others recounted incidents where despite being categorized as being at risk, they had to continue with their roles and provide care to sustain services. In the words of one participant:

*We normally run with a capacity of nine advanced clinical practitioners to cover this role but at the moment we only have two. That is myself as full time and my colleague as part time, so 1.5 really, so we are really short-staffed and we are a patient-facing team. We get a lot of pressure from the management and from the CCG* [Clinical Commissioning Group] *that we need to continue patient-facing irrespective of the challenges. So sometimes I’ve had to go against my risk assessment and go visit patients* [P19, Allied Health Professional].

Several participants also felt that their desire to practice safely and ethically was continually challenged by the reality of the staffing situation in the wards and they felt torn between their duty to save lives and adhere to strict COVID protocols. As one participant expressed:

*When you’re in a cubicle and ventilating a patient, somebody will scream “Help! Help!,” so you just run and you’re thinking risk assessment, I’ve been in with a COVID patient, I’m ventilating this patient but someone’s shouting help, a cardiac arrest, so you just run. There’s lots of breaches in there but then again the question is ethics versus morale versus what is right from wrong. Should I do this? Should I do an AGP* [Aerosol Generating Procedure] *in the middle of the corridor? If I don’t, this patient will die. Should I resuscitate and start compressions? If I don’t, they will die, but I’m in here in close contact and here comes the virus spreading all over the corridor* [P2, Nurse/Midwife].

### Risk Management and Risk Mitigation: Individual Responses

Participants described a range of measures, which they adopted at a personal level to protect themselves. These included changes in their routines both inside and outside the workspace, adjusting lifestyle habits, and changing living conditions with family. For instance, to prevent further exposure to themselves and to feel safer, some participants limited their use of public transport, using private cars were able. Most participants were extra attentive to personal hygiene and sanitization, mainly after finishing work, and ensured that they washed or changed out of their work clothes before entering the house. In the words of a participant:

*I think the main overriding theme was to ensure that when I left work every day, having worked on a COVID ward for 8 h or 12 h, that I certainly “de-COVI-fied” in my room, in my office, and then went home. So it was just mainly about ensuring that the transmission to immediate family was minimized* [P4, Doctor].

Some participants even went to great lengths to protect their vulnerable family members and resorted to measures such as moving out of their homes and staying in hotels and apartments to prevent transmission. One participant who did this shared:

*I have two family members who are shielding*…*so when COVID struck, I moved out into an apartment for 3 months whilst I was working on the ward because the ward did have COVID patients and we had a lot of staff off, so as not to put my family at risk* [P15, Ancillary Health Worker].

Caution was exercised in other areas of life as well, and social restrictions were diligently followed:

*I know other* [places of worship] *have opened up, but we’ve decided no we’re not going to open our* [place of worship] *and we’ve continued to keep social distance really and I mean absence distancing* [if] *that’s a word!* [P21, Ancillary Health Worker],

To reduce risk and to feel protected, many HCWs also decided to purchase their own PPE when things were in short supply. As one participant said:

*I wasn’t necessarily going to wait for the right things to be delivered into the right place, and some of us sort of took things into our own hands, and possibly I’m very grateful that I did do that given how things have panned out 9 months later. There was certainly a trend after that for some people investing in some things in addition to what was being provided on the wards* [P4, Doctor].

Some participants also described advocating for or making necessary arrangements for other vulnerable members in their teams. In this regard, a participant shared:

*They are still redeploying people who may be vulnerable into areas that they really shouldn’t be in—I had to speak to a colleague of mine saying “you really shouldn’t be there” because she had an elderly parent who had severe mental health problems and a son as well, and she still wasn’t recovered from pneumonia. So it was only then that she went onto sick leave. She had to go to her GP* [General Practitioner] [P6, Allied Health Professional].

## Discussion

In this nationwide study of HCWs, we undertook an in-depth exploration of HCWs’ experiences, fears, concerns, and perceptions about safety and protection, while working during the pandemic. The findings from this research generally align with the existing literature on United Kingdom healthcare organizations’ risk response during COVID-19. Our finding of HCWs experiencing PPE shortage, particularly during the first wave of the pandemic, has also been reported from the United Kingdom-REACH survey, where two-thirds of respondents said that they lacked appropriate PPE during the first lockdown (20). Risks to HCWs due to PPE shortage, frequently changing and inconsistent guidelines, PPE rationing, and poor quality PPE were also reported by other studies (13).

While these issues had affected many HCWs, a worrying perspective is the large number of ethnic minority staff who reported these issues. These reports align with previously expressed concerns about institutional racism impacting the inequitable access to appropriate PPE for ethnic minority nurses (5). Further complicating the risk of exposure, when ethnic minority HCWs did have access to PPE, design and implementation issues such as the failure of respiratory mask-fitting had further disadvantaged them (5). The issues of ethnicity in relation to risk and perceived risk are complex. One example of this is how ethnic minority participants indicated that they believed they would be at higher risk of the severe disease once they were infected, rather than at high risk of infection. Previous researchers have shown that the risk is probably mainly due to a higher risk of infection due to contributory factors such as PPE, but it could be that government communications, in the first wave of the pandemic, were lacking on this issue (21). Therefore, it is important to communicate the complexity of these issues to ethnic minority HCWs so they do not believe that they are inherently more vulnerable to COVID-19 than their White colleagues (22).

With regard to risk assessments, participants in this study reported perceptions of lack of adequacy as well as insincerity of intent (described as tick-box exercises) making them feel concerned for their own health and generating skepticism about whether their wellbeing was a priority for their employers (23). This perception could create mistrust in employers and also overall in healthcare organizations, institutions, and authorities. Lack of trust in employers puts an additional psychological burden on certain groups of HCWs (24), impacting their mental health and wellbeing at a time when they already facing increased psychological pressures, such as being exposed to higher levels of patient death than usual (25).

Previous research has suggested that, during the pandemic, bullying or prejudicial behavior has manifested itself *via* the redeployment of ethnic minority staff to COVID-19 “hot wards” increasing the risk of exposure (26, 27). This is significant for our findings as our participants also reported unsafe practices with regards to infection risk and clinical care from what they perceived as inappropriate redeployment which left them torn between their duty to save lives and adhere to strict COVID protocols. Furthermore, the national Workforce Race Equality Standard (WRES) survey has consistently identified a wider pattern of ethnic minority staff reporting harassment or bullying more at work as compared with their White colleagues (28).

Pressure to deliver care underpinned decisions taken by HCWs at all grades to continue working in environments they did not feel safe or protected within. Facing infectious disease outbreaks is neither novel nor unexpected for HCWs, but the scale of the outbreak coupled with issues around insufficient organizational protection provided a starkly different experience. Walking the tightrope between staff-patient obligations, staff personal risk affected clinical decision-making as a result, which in turn posed new questions around clinical ethics and practice described by the study participants (29).

Workforce planning issues arising from increased staff shortages, which pre-dated the pandemic, burgeoned during the pandemic due to sickness and redeployment. This not only increased the pressure to deliver on the few remaining qualified individuals but also heightened the risks that redeployed, and regular staff placed themselves at when attending to patients. This was in the context of the government’s strategy to protect the NHS and keep it operating at a certain capacity at all costs and at all times (30, 31), which put further pressure on the remaining workforce to continue with an “obligation to treat” even at great personal costs (29, 32).

This study has evidenced that individual members of staff employed their own risk mitigation strategies to protect themselves, their families, and the public. These included changes in their routines both inside and outside the workspace (such as reducing the use of public transport; washing and changing clothes when entering their homes and purchasing their own PPE), adjusting lifestyle habits (such as extra attention to personal hygiene and sanitization), and changing living conditions (such as moving out of their family home). It is clear that behavioral changes were expected of everyone. However, HCWs’ perception that the protective measures put in place for them were inadequate or insincere resulted in a perceived onus on them to look after their own wellbeing. Staff safety is an employer’s responsibility, and a situation where HCWs were made to take their safety into their own hands must not be allowed to persist. This needs to happen for responses to the current and future crises.

## Conclusion

Healthcare workers identified several risk areas and/or negative experiences of risk management during the pandemic. Our findings indicate that there have been several shortfalls at the organizational level in protecting HCWs. The pressure to keep the health system afloat came at great personal costs for many HCWs, which added to their emotional and mental burden. Ethnic minority HCWs also perceived greater risks, indicating institutional and structural racism, which has led to further loss of trust on employers. The heightened risks and perceived lack of sincerity on the part of employing organizations could have implications on staff retention, wellbeing, and safety, going forward. These findings point to the need for more robust inclusive and equitable risk assessment and mitigation processes, as well as transparent communication.

## Limitations

Our study had some limitations. This was a qualitative piece of research, and as such was not intended to provide quantitative data-driven comparisons of different experiences of risk by the HCW ethnic group. Rather, this research provides an insight into the lived experiences of a range of HCWs from an ethnically diverse workforce. Due to social distancing measures in place at the time, recruitment strategies and data collection had to be conducted remotely and using online technology. This may have affected participation from certain groups who may be less proficient in the use of or have less access to digital technology. Related to this, the number of HCWs in non-clinical and low-paid roles such as porters and domestic staff were limited in our study, which means that the risks unique to these roles may have not been adequately captured, and further research on these HCWs is needed to highlight the differential and distinctive risks faced by them. The relatively small sample size and the majority of data being from England may be a barrier to drawing conclusions about the entirety of the United Kingdom. In addition, the size of sample combined with the use of purposive sampling increases the chances that findings may have been affected by researcher and sampling biases.

## Data Availability Statement

The raw data supporting the conclusions of this article will be made available by the authors, without undue reservation.

## Ethics Statement

The study received ethical approval from the London-Brighton and Sussex Research Ethics Committee of the Health Research Authority (Ref No: 20/HRA/4718). The patients/participants provided their written informed consent to participate in this study.

## Members of United Kingdom-Reach Collaborative Group

Manish Pareek (Chief investigator), Laura Gray (University of Leicester), Laura Nellums (University of Nottingham), Anna L. Guyatt (University of Leicester), Catherine Johns (University of Leicester), I Chris McManus (University College London), Katherine Woolf (University College London), Ibrahim Abubakar (University College London), Amit Gupta (Oxford University Hospitals), Keith R Abrams (University of York), Martin D Tobin (University of Leicester), Louise Wain (University of Leicester), Sue Carr (University Hospital Leicester), Edward Dove (University of Edinburgh), Kamlesh Khunti (University of Leicester), David Ford (University of Swansea), Robert Free (University of Leicester).

## Author Contributions

All authors listed have made a substantial, direct, and intellectual contribution to the work, and approved it for publication.

## Conflict of Interest

The authors declare that the research was conducted in the absence of any commercial or financial relationships that could be construed as a potential conflict of interest. MP reports grants from Sanofi, grants and personal fees from Gilead Sciences and personal fees from QIAGEN, outside the submitted work.

## Publisher’s Note

All claims expressed in this article are solely those of the authors and do not necessarily represent those of their affiliated organizations, or those of the publisher, the editors and the reviewers. Any product that may be evaluated in this article, or claim that may be made by its manufacturer, is not guaranteed or endorsed by the publisher.
