# Supplementary figures and images for: Healthcare Workers From Diverse Ethnicities and Their Perceptions of Risk and Experiences of Risk Management During the COVID-19 Pandemic: Qualitative Insights From the United Kingdom-REACH Study
Source: Front Med (Lausanne). 2022 Jul 1;9:930904. doi: 10.3389/fmed.2022.930904 (PMC9285103; doi:10.3389/fmed.2022.930904)

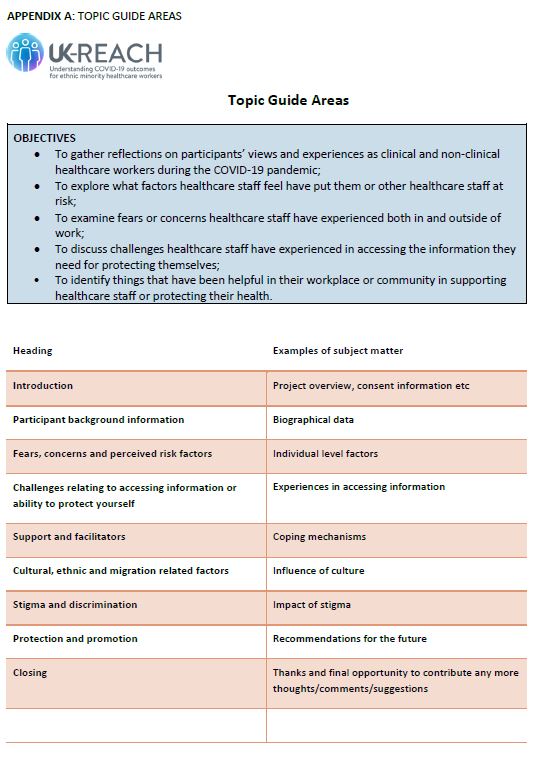

Supplement: Supplementary file 1 [file Image_1.jpg]
